# Microbial degradation of citrate mediates sealing of cement cracks under anaerobic conditions relevant to radioactive waste disposal

**DOI:** 10.1038/s41529-025-00686-4

**Published:** 2025-11-20

**Authors:** Natalie Byrd, Ananya Singh, Naji M. Bassil, Joe S. Small, Frank Taylor, Christopher Boothman, Dirk L. Engelberg, Sultan Mahmood, Tristan Lowe, Jonathan R. Lloyd, Katherine Morris

**Affiliations:** 1 Radioactive waste Disposal and Environmental Remediation National Nuclear User Facility and Williamson Research Facility, Department of Earth and Environmental Sciences, The University of Manchester, Manchester, UK; 2 Nuclear Waste Services, Pelham House, Seascale, Cumbria, UK; 3 Metallurgy & Corrosion, Department of Materials, The University of Manchester, Manchester, UK; 4 Manchester X-ray Imaging Facility, Photon Science Institute, University of Manchester, Manchester, UK; 5 Natural History Museum Abu Dhabi, Jacques Chirac Street, Abu Dhabi, UAE

**Keywords:** Environmental sciences, Environmental chemistry, Biomineralization

## Abstract

In radioactive waste repositories, cement is used for construction, backfill, and waste encapsulation. Over time, cracks may form, creating potential pathways for contaminant migration. A self-sealing mechanism is through calcium carbonate (CaCO_3_) precipitation, which can be driven by microbial oxidation of organic compounds. We explored microbially induced calcite precipitation facilitated by metabolism of citrate, a complexant in low- and intermediate- level radioactive waste (L/ILW). Nitrate-reducing microcosms containing cement pellets, citrate, nitrate, alkaline sediment inoculum, and synthetic groundwater (pH 11.2) were incubated in the dark (20 °C, 40 days). Aqueous geochemical data revealed complete citrate removal, denitrification, pH decrease to pH 9, and removal of Ca^2+^_(aq)_. Furthermore, 16S rRNA gene sequencing showed enrichment of citrate-oxidising/nitrate-reducing bacteria. Solid phase analysis (XRD, SEM-EDS, µXCT) confirmed new calcite precipitates reduced cement porosity and sealed cracks at the surface. Overall, microbial oxidation of organic ligands under alkaline conditions may reduce contaminant mobility in L/ILW repositories through calcite precipitation and crack sealing.

## Introduction

Low- and intermediate-level radioactive wastes (L/ILW) represent the largest volumes of radioactive waste inventories worldwide^1^. Both L/ILW are highly heterogeneous, containing radionuclides, heavy metals, and organic complexants including decontamination agents such as citric acid^1^. In the UK and other nuclear nations (e.g., France, USA, Sweden), disposal concepts for L/ILW are specially engineered repositories designed with a multibarrier system aiming to contain waste and metal/radionuclide contaminants within acceptable levels, e.g., the UK’s Low Level Waste Repository in West Cumbria^1,2^. Cement typically plays a critical role in the engineering design of these repositories, serving as a key material for construction, backfill, and waste encapsulation^1–3^. Cementitious materials are also anticipated to strongly influence the biogeochemical processes within repositories, as alkaline pore waters are generated during re-saturation of post-closure facilities^4,5^. Indeed, over centuries post-closure, the biogeochemical conditions within repositories for L/ILW are expected to evolve as the interactions between the surrounding far field environment, microorganisms, waste components, and structural materials, like cement, impact the disposal environment^5,6^. Despite the alkaline conditions expected to develop in L/ILW repositories, microbial life is unlikely to be eliminated; specialized microorganisms have thrived in analogous high pH systems for centuries (e.g., Maraqin, Jordan; Harpur Hill, UK; and the Semail Ophiolite, Oman)^7,8^. Additionally, previous research highlights anaerobic biogeochemical processes occur under the elevated pH conditions (pH 10–12) expected in cement environments, e.g., nitrate, Fe(III), and radionuclide (e.g., U, Np, Tc) bioreduction, as well as the biodegradation of organic compounds including organic complexants such as isosaccharinic acid and citrate^9–15^. These processes are expected to enhance contaminant retention and contribute to an additional ‘bio-barrier’ within the repository system^5,6^.

Understanding microbial impacts on multibarrier components like cement underpins repository design, operation and environmental safety case development. More broadly, new insights are of interest in bioremediation of cementitious contaminated land or to the wider engineering community where cement is ubiquitous^16–21^. In L/ILW repositories, cement is susceptible to physico-chemical alteration (e.g., cracking and carbonation) over time which may impact functions that prevent contaminant migration, e.g., by altering structural integrity^22^. Microbes can catalyse reactions that drive physico-chemical alteration of cement e.g., decalcification via leaching, carbonation, and sulfate attack, altering porosity, structural integrity, and porewater pH^23,24^. Of these, carbonation, including microbially induced carbonate precipitation, may be favourable as it can promote self-healing of voids and cracks^25,26^. Thus, carbonation can close off pathways for contaminant migration and/or aid in precipitation of contaminant-bearing mineral phases e.g. ^90^Sr-substituted CaCO_3_^27^. In alkaline environments, carbonation occurs when carbonate or bicarbonate species (from equilibration of dissolved CO_2_) react with calcium-rich cement phases (e.g., portlandite, calcium silicate hydrate), leading to oversaturation and precipitation of calcium carbonate minerals and leading to precipitation in and sealing of cracks and voids^26^. Bacterial oxidation of organic carbon (e.g. citrate) under anaerobic (e.g. nitrate-reducing) conditions, generates CO_2_ which has been shown to enhance precipitation of calcite and carbonate minerals^15,28^.

In L/ILW repositories, high organic carbon wastes can provide substrates from which microbes can produce CO_2_ and, accordingly, microbial degradation has potential to enhance carbonate precipitation under the high Ca and pH conditions expected in the wastes^18,26,29^. Complexing agents used in decontamination are sources of organic carbon in L/ILW wastes, with citrate being a primary decontaminant that forms stable complexes with metals and radionuclides^30–33^. Experiments at pH 10–11 have demonstrated that the oxidation of organics, including citrate, under nitrate-reducing conditions can increase levels of dissolved CO_2_ and lead to carbonic acid induced acidification of systems^12–15,29,34–39^. Despite this, there is a paucity of information on citrate biodegradation in the presence of cementitious materials especially with respect to understanding impacts on calcium carbonate mineralisation. Clearly, degradation of citrate and other organic complexants in L/ILW waste is desirable for preventing metal contaminant solubilisation. Citrate biodegradation in a cementitious system, and the resultant CO_2_ that forms, could also be beneficial in terms of driving carbonation processes and sealing cracks/sequestering radionuclides such as ^90^Sr through calcite formation^27,40,41^. The current work aims to understand anaerobic citrate biodegradation in a cementitious system and under nitrate-reducing conditions, with a particular focus on exploring impacts on cement integrity from microbially-induced carbonation reactions.

## Results

Batch microcosm experiments, containing cement pellets (3:1 ratio of pulverised fly ash [PFA] to ordinary portland cement [OPC]) and a high pH microbial inoculum were set up and monitored over 40 days. Microcosms were amended with citrate and nitrate, to stimulate nitrate bioreduction, with no-citrate controls run in parallel. Periodic sampling and analysis of aqueous (IC, ICP-AES, pH), solid (XRD, SEM-EDS, XCT) and microbial (16S rRNA gene sequencing) components of these experiments was conducted, supplemented with geochemical modelling (PHREEQC, ThermoChimie version 12a).

### Aqueous geochemical analysis: citrate biodegradation in the presence of cement

**Fig. 1:**
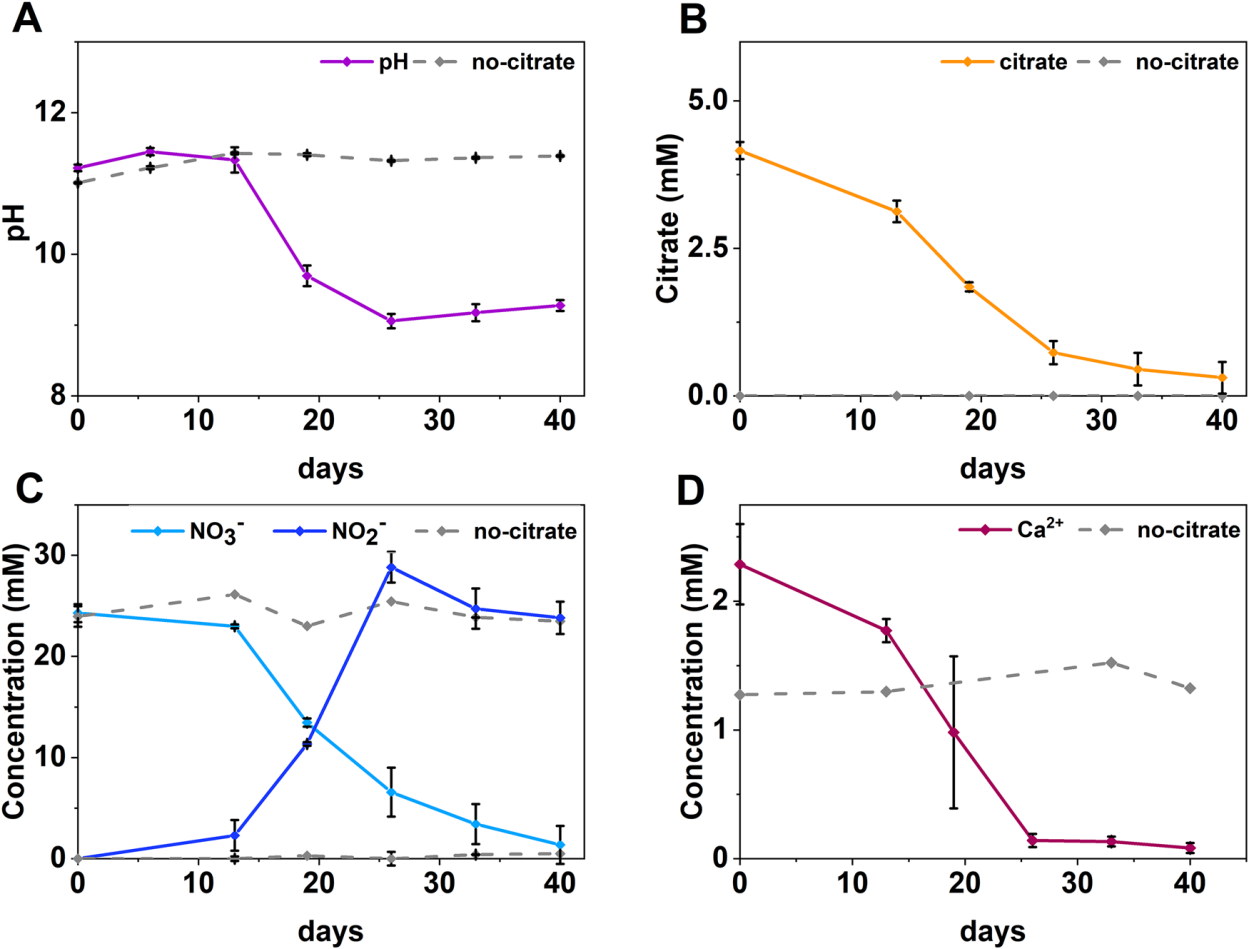
Aqueous geochemical data from citrate-supplemented and no-citrate control microcosms. Panels show: (**A**) pH, (**B**) citrate concentration, (**C**) nitrate/nitrite concentration and (**D**) Ca^2+^(aq) concentration. Error bars represent 1σ on triplicate measurements. The solid colour lines represent citrate-treated microcosms and the dashed grey lines represent the no-citrate control microcosms.

Denitrifying sediment microcosm experiments were set up to explore citrate biodegradation under elevated pH conditions, in the presence of cement pellets. In citrate-supplemented microcosms, citrate degradation started by day 13, and was essentially complete by day 40 (3.9 ± 0.3 mM citrate was removed; Fig. 1B). No volatile fatty acids (VFAs) were detected, and the pH dropped from 11.2 to 9.1 by day 26, indicating oxidation of citrate to CO_2_ and acidification via carbonic acid formation (Fig. 1A). Citrate oxidation was coupled to nitrate reduction; by day 13, measurable nitrate removal and nitrite ingrowth had initiated in the citrate supplemented microcosms (Fig. 1C). By day 40, essentially complete removal of nitrate (28.8 ± 2.0 mM nitrate removed) had occurred. Nitrate was reduced to nitrite (22.9 ± 1.5 mM at day 26), which underwent further reduction by day 40 (5.0 ± 2.1 mM nitrite reduced by day 40). Electron balance calculations from geochemical data (Fig. 1A–C) and standard electron equivalents (Table S1)^42^ showed that citrate oxidation (3.9 ± 0.3 mM) generated approximately 70 ± 5.4 mM electron equivalents, while total denitrification (22.9 ± 2.0 mM nitrate and 5.0 ± 2.1 mM nitrite) consumed approximately 61 ± 7.4 mM electron equivalents. This suggested stoichiometric balance to support denitrification was occurring. No changes in aqueous geochemistry were observed in the parallel no electron donor and sterile controls (Fig. S1) controls confirming that microbial metabolism of citrate was driving denitrification.

To investigate whether metabolites from microbial citrate oxidation were promoting the precipitation of CaCO_3_ phases, via production of CO_2 (aq)_, Ca^2+^_(aq)_ concentrations were monitored by inductively coupled plasma – atomic emission spectroscopy (ICP-AES). The initial Ca^2+^_(aq)_ concentration in citrate-supplemented microcosms was 2.3 mM and 96% Ca^2+^_(aq)_ removal was measured by day 40 (Fig. 1D). The Ca^2+^_(aq)_ removal occurred over the same timescale as citrate biodegradation and nitrate reduction (Fig. 1A–C), suggesting Ca^2+^_(aq)_ was reacting with the metabolites produced, likely bicarbonate at pH 9. This is supported by geochemical modelling (PHREEQC with ThermoChimie version 12 A) of citrate-amended microcosms, using measurements from ICP-AES, ion chromatography (IC) and pH analysis. This indicated that CO_2 (aq)_ generated from citrate oxidation would equilibrate as carbonate and lead to oversaturation and precipitation of CaCO_3 (s)_ under the relevant experimental conditions (saturation index = 4.4; Fig. S2, S3). In contrast, Ca^2+^_(aq)_ levels in the no citrate control incubations were stable over the duration of the experiment (Fig. 1A–C) and emphasising that microbial metabolism of citrate by nitrate-reducing bacteria was driving Ca^2+^_(aq)_ removal through calcite formation. Overall, there were higher total Ca^2+^_(aq)_ levels in citrate supplemented microcosms, compared to the no citrate controls, which is likely due to Ca-citrate complexation (Fig. S2).

### Solid phase analysis of cement pellets after citrate biodegradation

**Fig. 2:**
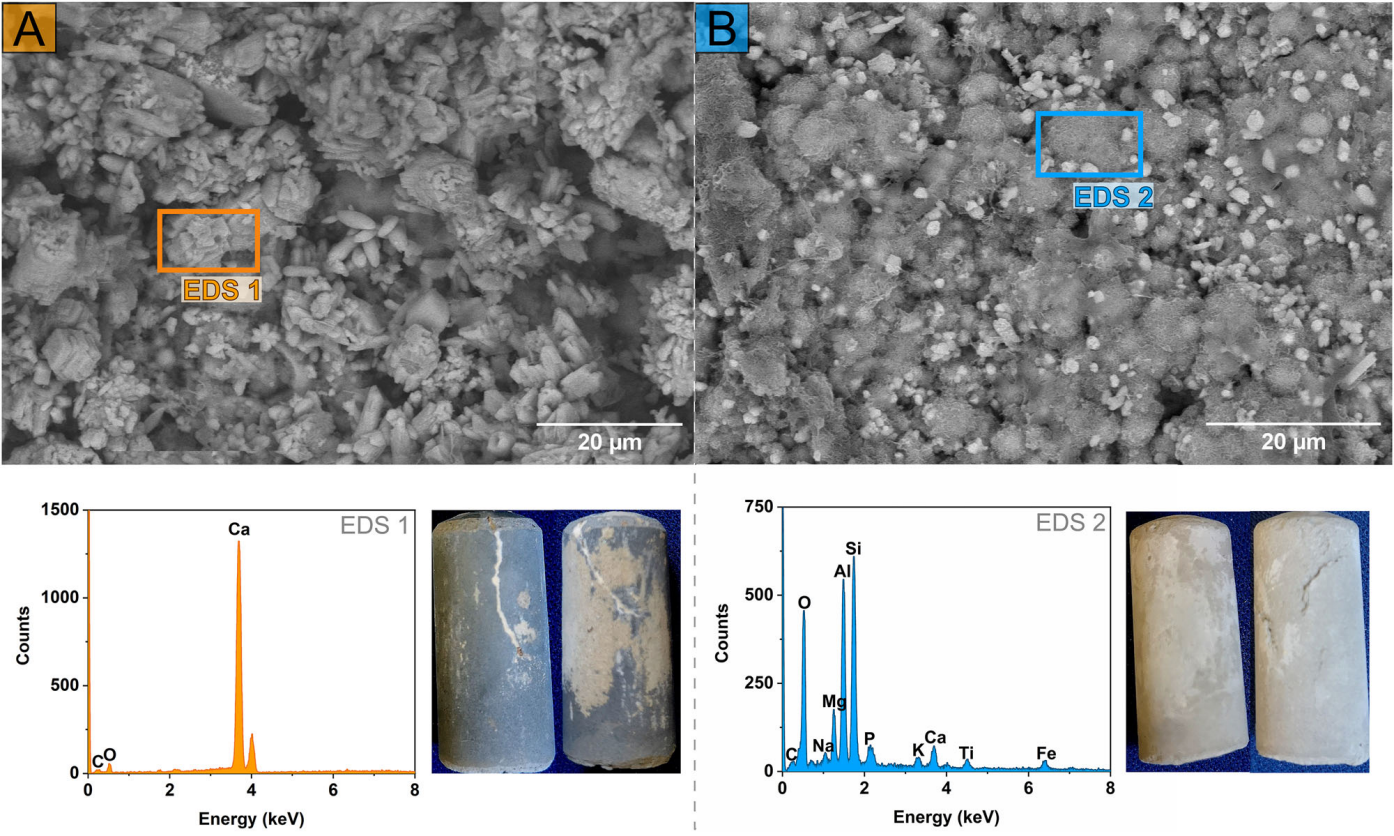
SEM images (top), corresponding EDS and camera images (bottom, inset) of a cement pellet from. **A** The citrate-supplemented microcosms and (**B**) no-citrate control microcosm. The coloured rectangles on the SEM images indicate the area where the EDS was measured and are representative across samples.

Visual inspection of the cement pellets extracted from citrate-supplemented microcosms after the experimental endpoint (day 40) revealed a pale mineral coating had formed on the surface, consistent with calcite deposition (Fig. 2), as predicted by PHREEQC modelling (Fig. S2 and S3). Conversely, cement pellets from no electron donor controls lacked this pale coating. Cement pellets from both systems underwent characterisation by scanning electron microscopy (SEM), x-ray diffraction (XRD) and x-ray computed tomography (XCT). First, SEM imaging of pellets from the citrate-supplemented microcosms showed calcite-like, rhombohedral morphology on the surface Fig. 2^43,44^. Corresponding energy dispersive X-ray spectroscopy (EDS) analysis showed consistent strong signals across the sample for co-located Ca, C, and O (Fig. 2A; Fig. S7), and XRD analysis of surface precipitates on the samples (selectively sampled from the cement pellet surface) confirmed that this was calcite (Fig. S4). By contrast, the cement pellet from the parallel no electron donor (no citrate) control microcosm did not have the same pale precipitates on the surface, and SEM imaging showed features that were predominantly calcium silicate hydrate-like^45,46^, with relatively small crystalline inclusions. Corresponding EDS analysis of this pellet consistently detected a wider variety of elements from different locations measured on the pellet surface including Al, Si, Fe, Ca, Mg, C and O (Fig. 2B, Fig. S7); no surface precipitates could be sampled for XRD analysis. Surface morphologies and elements identified by EDS in the control are consistent with fresh cement pellets (Fig. S5 and S6), and cement in general, both being rich in silica and aluminium oxides^47,48^. PHREEQC modelling (Fig. S3), based on day 40 geochemical data, also predicted oversaturation with respect to other calcium-bearing phases, such as calcium silicate hydrate (e.g. CSH0.8 SI = 0.2; Fig. S3). However, the actual precipitation of these phases appears to have been minimal, if it occurred at all. No calcium silicate hydrate gels were observed in SEM images of samples from citrate microcosms (Fig. 2), and no crystalline Ca-bearing phases were detected by XRD analysis of surface precipitates (Fig. S4).

**Fig. 3:**
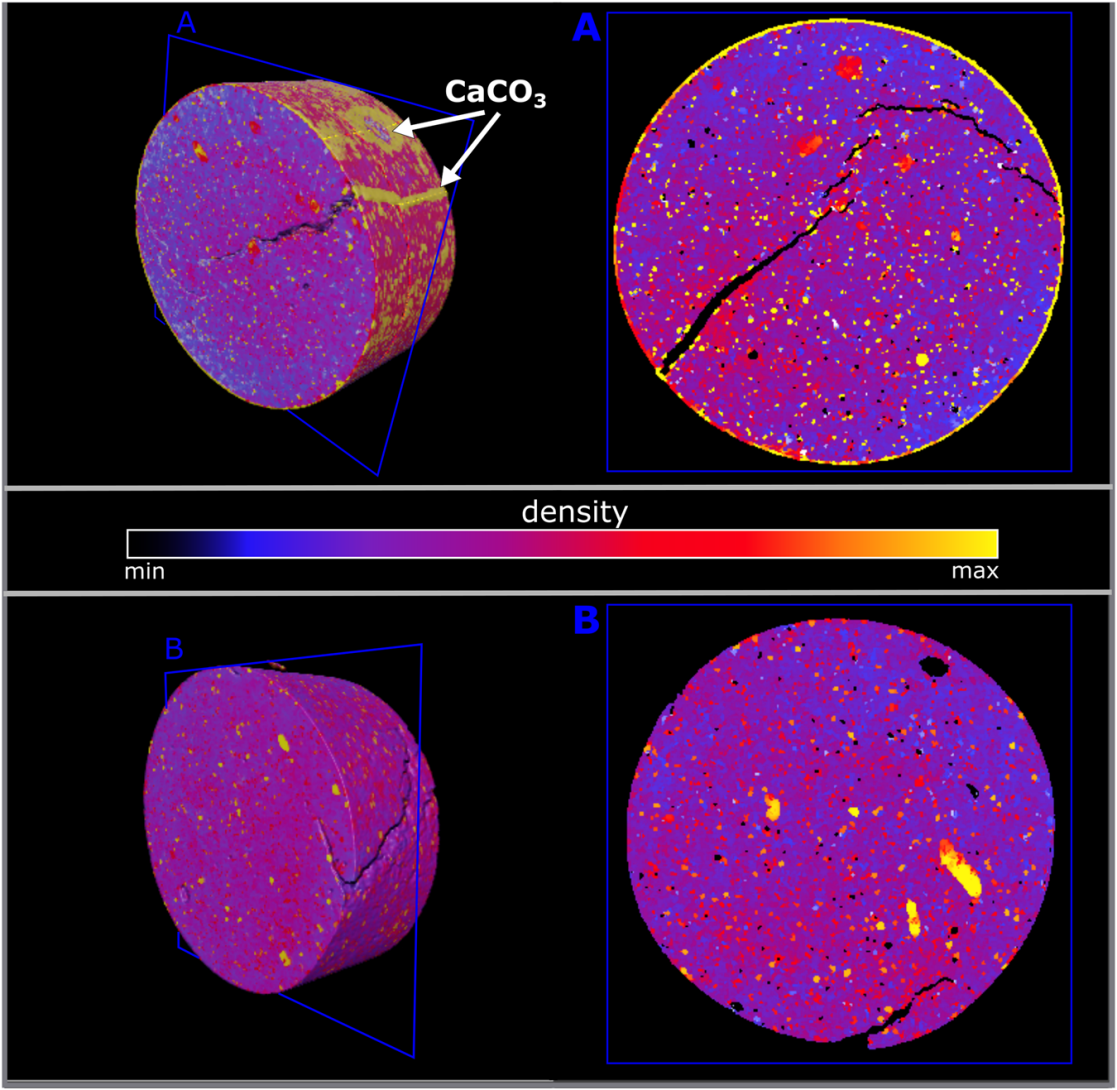
XCT reconstructed 3D visualisations (left) and cross sections of cement pellet samples (right) from the citrate-supplemented microcosms. (**A**) and no electron donor (no citrate) control microcosms (**B**). The blue box indicates the location of the cross section within the 3D sample volume. The scale bar represents the relative density of phases in the cement; based on XRD (Fig. S4) the yellow ‘halo’ on the outside of the sample from the citrate-supplemented microcosm (**A**), represents calcite. The brightness intensity of the raw greyscale slice images has been converted to the uniform colour map, ‘thermal’ to aid in visualisation of features (Crameri et al. 2020).

To further understand the extent of the calcite (bio)precipitation, 3D-XCT analysis of selected cement pellets (representative of the triplicate set; Fig. S10) from the citrate-supplemented (Fig. 3A) and no-citrate microcosms (Fig. 3B) was performed. 3D reconstructions of images revealed cracks (approximately 1–5 mm long, 0.1–0.8 mm wide) visible within both samples. As expected, pellets from citrate-supplement experiments exhibited clear deposition of an X-ray attenuating material on the surface, which infilled crack openings (Fig. 3A). The distribution of this material (Fig. 3A) corresponds to the location of visible precipitates on the cement pellet surface (Fig. 2A) already identified as calcite by SEM and XRD (Fig. 2, Fig. S4). Accordingly, the 3D XCT images (Fig. 3A) suggest pale calcite deposits at the cement pellet surface had sealed cracks and infilled surface-voids in citrate supplemented experiments. Clearly, this had not occurred in the no citrate controls (Fig. 3B). Grains of x-ray attenuating phases are visible within both cement samples (Fig. 3A, B), presumably these are also calcium carbonate phases, known to form during cement hydration^49^.

**Fig. 4:**
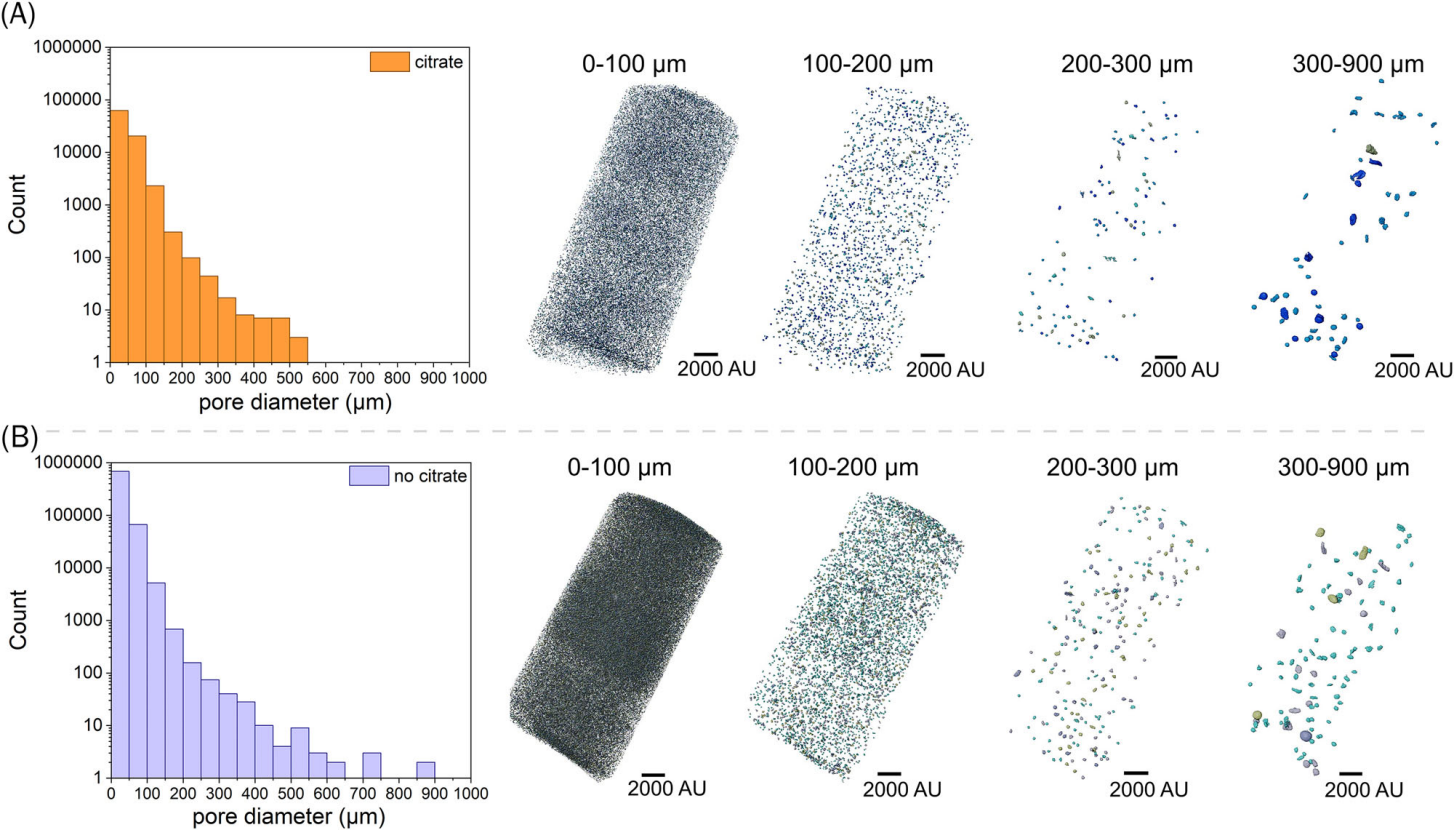
Pore size distribution from segmentation and pore size analysis of 3D volumes obtained from XCT imaging of cement pellets extracted from. **A** A citrate-amended microcosm, and (**B**) a parallel no-citrate control microcosm (histogram bin size = 15 μm). The scan resolution was 5 μm, establishing the technical limit of spatial resolution in the dataset. The minimum voxels required for reliable pore identification was three voxels, setting the confident detection threshold at 15 μm. Pores smaller than this threshold (<15 μm) were excluded from this analysis.

The extent of microbially mediated calcite deposition on cement pellets was further assessed using segmentation and pore size analysis of 3D volumes obtained from XCT. All cement pellet samples were 2.5 cm long with 1 cm diameters; thus, the volume of each pellet was 2 × 10^12^ µm^3^. The total number of pores identified for cement pellets from citrate treated systems was approximately 9 × 10^4^ pores, significantly fewer than in the no citrate system with 8 × 10^5^ pores (Fig. 4). Accordingly, cement pellets from citrate microcosms were calculated to have reduced total pore volume (1 × 10^10^ µm3; 0.5% porosity), compared to the no citrate controls (3 × 10^10^ µm^3^; 1.5% porosity). This suggests that for citrate stimulated experiments, microbially mediated calcite precipitation was reducing the total void space in the cement. Interestingly, the average pore diameter in cement from citrate experiments (42 µm) was significantly (*P* < 0.0001) larger than in no citrate controls (29 µm). Considering this, alongside the measured reduction in pore volume, suggests that calcite preferentially precipitated in smaller voids until they were either completely filled or no longer detectable by XCT. Indeed, 99% of cement pores were 0–100 µm in diameter (the smallest size range) in the no citrate system, vs 96% for cement from citrate-treated systems. Additionally, pore size distributions (Fig. 4) showed filling of very large pores, diameters >550 µm, in cement from the citrate system, consistent with visible infilling in 3D reconstructed images (Fig. 3).

### Microbial ecology

**Fig. 5:**
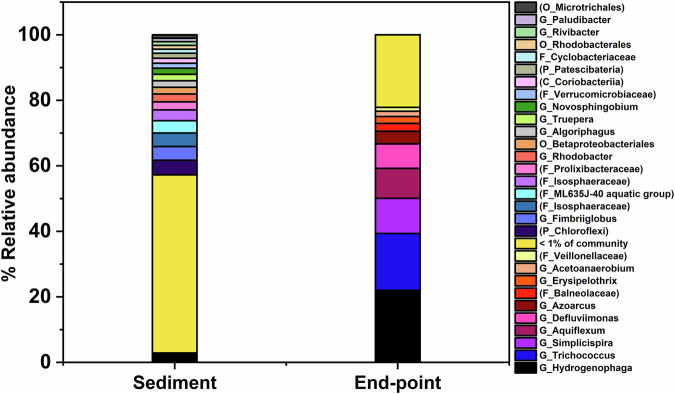
Data from 16S rRNA gene sequencing showing the microbial community at the genus level for the sediment inoculum at day 0 (left bar) and sample from the day 40 experimental endpoint (right bar). Where sequences could not be resolved at genus level, the highest possible taxonomic rank was resolved and is indicated in brackets e.g., ‘(O_Microtrichales)’ indicates this sequence could only be resolved at order level and was most closely matched to Microtrichales.

PCR-based high-throughput 16S rRNA gene sequencing was used to characterise communities in the initial high pH sediment inoculum and day 40 experimental endpoint slurry samples from the citrate-supplement microcosms. Attempts to extract DNA from cement surface were made but DNA yields were below the limit of detection by PCR and no signs of microbial colonisation of the surface could be observed by SEM imaging (Fig. 2, Fig. S7), or fluorescent microscopy imaging (Fig. S9). In the sediment inoculum 562 operational taxonomic units (OTUs) were detected, and by day 40 there was a measured decrease to 265 OTUs (alpha diversity plot in Fig. S8). The dominant phylogenetic classes detected in the starting inoculum were Bacteroidia (21%), Planctomycetacia (14%), and Alphaproteobacteria (13%). By the experimental endpoint, the community had shifted, and Gammaproteobacteria (39%) and Bacilli (18%) had become the dominant classes. Closer inspection of Gammaproteobacterial sequences (Fig. 5) in the day 40 sample identified several genera with members capable of citrate-oxidation and nitrate-reduction in the citrate-supplemented microcosms. The most dominant of these were: *Hydrogenophaga* (22% of sequences), *Simplicispira* (11%), *Aquiflexum* (9%), and *Defluviimonas* (7%)^50–53^. Bacteria affiliated with the genus *Hydrogenophaga* have previously been detected in samples from similar microcosm systems using the same sediment inoculum (e.g., ref. ^54^). Members of this genus can perform both organotrophic and hydrogenotrophic denitrification^55,56^. Closer inspection of the Bacilli sequences found they predominantly matched organisms affiliated with the genus *Trichococcus* (Fig. 5). Although members of the genus *Trichococcus* are typically fermentative, some species can reduce nitrate, oxidise citrate and grow at alkaline pH e.g., *Trichococcus flocculiformis*^57,58^; this organism was identified as one of several 100% species-level matches for this sequence.

## Discussion

In microcosms, microbial citrate oxidation was coupled to nitrate reduction at pH 11, in the presence of cement under conditions relevant to cementitious repositories for low- and intermediate-level radioactive waste disposal. In citrate-supplied microcosms, complete oxidation of citrate as the electron donor coupled to bioreduction of nitrate was demonstrated (Fig. 1), and this was supported by 16S rRNA gene sequencing which showed a pronounced enrichment in known citrate-oxidising and nitrate-reducing bacteria at the experimental endpoint (Fig. 5). The citrate oxidation/nitrate reduction mechanism reported here, in the presence of cement, was consistent with previously reported citrate oxidation/nitrate bioreduction reactions in non-cementitious microcosms at pH 10 and 11^15^. In fact, microbial nitrate reduction rates (0.9 mM day^−1^, this study) were slightly enhanced compared to those previously reported (0.5 mM day^−1^^15^) for pH 11 citrate oxidation/nitrate reduction. Furthermore, the rates for citrate oxidation (0.1 mM day^−1^_,_ this study) were essentially the same^15^. This demonstrates that the presence of cement does not impede microbial nitrate reduction or citrate biodegradation at pH 11 and that in high nitrate wastes containing suitable electron donors, this is likely to be an important pathway for removal of organic complexants in alkaline cementitious repositories^12,29,36,38^.

It should be noted that L/ILW repositories will likely have higher cement-to-solution ratios than the batch systems used here. The pH we targeted in our experiments (approximately pH 11) was based on geochemical modelling predictions for low level waste repository disposal conditions^59^. In systems with higher cement-to-solution ratios, there is potential for hyperalkaline pH (>pH 12) conditions to arise in the repository near-field which would likely inhibit microbial processes. In these scenarios, findings here would be most relevant for less alkaline (<pH 12) areas of L/ILW repositories, including at cement/air/sediment interfaces (i.e., within chemically disturbed zones) or in micro niches of lower pH arising due to e.g., waste heterogeneity. Nevertheless, these findings suggest organic complexants are unlikely to persist to the far field as biodegradation is likely to occur as the pH environment evolves to the disposal relevant, alkaline conditions explored here (e.g., ~pH 11). Findings here suggest rapid degradation occurs even in cementitious environments, likely rendering complexants short-lived relative to the multi-millennial lifespan of a repository.

Interestingly, 16S rRNA sequencing results from the current study (Fig. 5) indicated the bacterial community driving citrate oxidation/nitrate reduction in pH 11 microcosms with cement differed from those in similar high pH microcosms without cement^15^. This suggests that the presence of cement impacted the microbial community structure, although the reasons for this are unclear and require further investigation. Nonetheless, there was both a similar rate and extent of citrate degradation/nitrate reduction across both studies, and while cement may alter the microbial community composition, the functionality of the community (e.g., citrate biodegradation/nitrate-reduction) remained similar. This highlights multiple possible routes for high-pH anaerobic citrate biodegradation/nitrate reduction, which is perhaps unsurprising as this metabolic couple is highly energetically favourable even under extreme pH conditions >10 ^42,60^. The robustness of alkaliphilic citrate degraders is reassuring in the context of L/ILW disposal, as it seems likely microbes with this functionality will readily colonise heterogeneous, cementitious repositories given the appropriate electron donor and acceptors are present in the waste.

After geochemical analyses, corresponding results from solid phase analysis suggest that microbes had facilitated calcite precipitation on/in the cement. Data from SEM, XRD, and XCT analysis showed calcite precipitates coated surfaces of cement pellets from the citrate-supplemented microcosms (Figs. 2–4), whilst cement from no-citrate controls was largely unaltered compared to the starting material. Cement from citrate microcosms showed precipitates had sealed crack openings and filled pore spaces. Despite visible crack-sealing and pore filling in the XCT images, the void space within large cracks was not filled (Fig. 3). Reasons for this remain unclear, but likely explanations include; (a) substrate depletion prevented further reaction or, (b) calcite deposits sealed voids at the surface, preventing HCO_3_^−^ entering cracks to facilitate further calcite formation. Further work is needed to explore the impacts of microbially induced carbonation on fluid flow and contaminant retention (e.g., hydraulic conductivity tests of microcosm-treated samples) and to further understand how carbonation/crack-sealing develops in a full-scale, dynamic repository environment. Indeed, thermodynamic equilibrium modelling (e.g., 1D transport models) and/or flow-through experimental systems where substrates can be replenished and/or metabolites are transported are an obvious next step to fully translate these initial findings to larger scale scenarios. Additionally, solid phase analysis showed an overall reduction in pore volume in citrate stimulated experiments. This may have varied impacts in the context of a repository by restricting pathways for both gas diffusion and contaminant migration, understanding these impacts is another important aspect to consider in future work. Collectively, the data suggest that the biodegradation of citrate (and likely other organic electron donors) under nitrate-reducing conditions can mediate calcite formation in a L/ILW repository. This is likely a favourable outcome, both in terms of limiting pathways for aqueous contaminant migration and/or as newly formed minerals may promote radionuclide sorption/incorporation^61–64^. For example, precipitation of metal carbonate minerals is a well-demonstrated mechanism for the sequestration of radionuclides e.g., ^90^Sr^27,40,41^. While data from these batch systems provide a clear biogeochemical baseline, showing that microbial metabolism of organic ligands can drive calcite precipitation and measurably reduce cement pore volume, we highlight that further research focused on longer-term, more dynamic systems is now required to explore the impacts of the microbial metabolism of organic compounds in disposal-relevant conditions.

Overall, the findings here provide evidence that citrate, commonly used as a complexant in the nuclear industry, is likely to be biodegraded and removed in a cementitious L/ILW repository setting. In turn this generates bicarbonate which reacts with Ca^2+^ rich cement phases to precipitate calcite, a process which could mediate the sealing of cement cracks and pores under pH 11 conditions, most relevant at the cementitious interfaces between a repository and the environment. These findings underpin the development of safety cases for radioactive waste disposal and directly inform management of the UK’s LLW repository. More broadly, this study contributes to an improved understanding of the microbe-cement interface in high pH environments, relevant to construction and civil engineering. Future work should prioritise understanding interactions in longer-term dynamic, flow through microbe-cement systems and/or include L/ILW relevant contaminant metals and radionuclides.

## Methods

### Sediment

High pH, Ca-rich sediment inocula were collected from a well characterised site, Harpur Hill in Derbyshire, UK and stored in the dark at 4 °C prior to use in microcosm experiments. Sediments were typically used in microcosm experiments within 4 weeks of collection^29,65^. At the point of collection, the sediment and groundwater slurry had a pH of approximately 11.

#### Cement pellets

Cement pellets were made using a ratio of 3:1 pulverised fly ash (PFA) to Ordinary Portland Cement (OPC) reflecting the make-up of grout used in low level radioactive waste facilities. The OPC was sourced from Hanson (BS EN197-1 CEM 1 52,N) and the PFA from ScotAsh Ltd. To make the cement slurry, the dry ingredients were combined with 18 MΩ deionised water (solid to liquid ratio of 0.42). The cement slurry was cast into pellets using a mould which generated identical cylindrical pellets of 2.3 cm length and 1 cm diameter, and cured for 2 weeks. Cured cement pellets were rinsed by running under a continuous stream of deionised water for 2 minutes per pellet whilst gently agitating the surface and then soaked for at least one week in deionised water. This removes Na and K hydroxides, which would cause extreme hyperalkaline (>pH 12) conditions to develop^4,47^.

#### Microcosm set-up

Anaerobic microcosms were set up in triplicate (under N_2_ atmosphere, by purging bottles with pure N_2_ for 1 hour), containing: 100 mL synthetic groundwater (Supplementary Information, Table S2)^66^, 5 g sediment inoculum, 5 mM trisodium citrate as electron donor, 30 mM NaNO_3_ as electron acceptor, and a 3 g cement pellet. To ensure the experiment started at pH 11 the cement pellet and sterile groundwater were added to the serum bottles and stored in the dark until the pH was approximately 11 (this took approximately 7 days). Subsequently, the sediment inoculum, citrate, and nitrate (both filter sterilised) were then added to initiate the bio-reduction experiment, which was incubated for 40 days. No electron donor controls (i.e., no citrate), sterile (autoclaved), and no sediment controls were set up in parallel (Fig. S11 illustrates the microcosm set up).

#### Geochemical analysis

Sediment slurry was periodically extracted under anaerobic conditions. The pH and Eh were measured using a Denver Instrument digital meter and a Fisherbrand FB68801 electrode, calibrated before measuring each time point using pH 7, 10 and 12 buffers (Fisher Scientific). Samples were centrifuged at 14,800 g for 5 min to remove solids. Supernatant was analysed by ion chromatography (IC; citrate, VFAs, NO_3_^−^, NO_2_^−^) and Inductively Coupled Plasma Atomic Emission Spectroscopy (ICP-AES; Ca^2+^) using a Dionex ICS5000 ion chromatography system and Perkin-Elmer Optima 5300 DV ICP-AES. Geochemical modelling using PHREEQC^67^, with the ThermoChimie database (version 11a^68^), was used to aid interpretation of data.

#### X-ray Diffraction (XRD)

Unaltered cement pellets and post-bioreduction mineral precipitates which formed on the surfaces of the pellets were analysed by XRD. The unaltered pellet was ground into a fine powder, and the post-bioreduction precipitates were carefully removed from the cement surface with a stainless steel spatula. Powdered samples were mounted on a quartz XRD slide and analysed using a Bruker D8 Advance. XRD conditions were as follows: Cu K α 1 X-rays at 5-70 degrees, 0.02 degree step size at 0.5 second per step. Crystal patterns were matched using Eva v14 against standards from the International Centre for Diffraction Data database.

#### Scanning Electron Microscopy (SEM)

Post-bioreduction, cement pellets were analysed by environmental scanning electron microscopy (ESEM). Pellets were removed from microcosm bottles in the anaerobic cabinet and gently rinsed using deionised water to remove any precipitates not adhered to the surface. To preserve microbial cells adhered to their surface, cement pellets were fixed using glutaraldehyde (2.5% glutaraldehyde in phosphate-buffered saline (PBS) solution) and dehydration with graded ethanol (25, 30, 40, 50, 60, 70, 80, 90, 100%; Newsome et al. 2018). Dried samples were then gold-coated and imaged using a FEI XL30 environmental scanning electron microscope (ESEM) equipped with a field emission gun (FEG), operating in 15 kV in high vacuum mode (10^-3^ to 10^-5^ mbar). Energy dispersive X-ray analysis (EDS) was performed using the EDAX Gemini EDS system.

#### X-ray Computed Tomography (XCT)

Dried cement pellets extracted from bioreduced microcosms and no-citrate controls for SEM analysis were also analysed by X-ray Computed Tomography (XCT). The pellets were scanned at 110 kV, 91 µA using a Nikon XTEK 224 keV X-ray Computer Tomography system housed within a customised bay fitted with a Perkin & Elmer 1611 detector, at the Henry Moseley X-ray Imaging Facility, University of Manchester. The large datasets (2000 slice images) obtained from scanning the cement samples were trimmed to a more manageable size (275 image slices per sample) and cropped using FIJI (https://imagej.net/Fiji) before filtering and segmentation in Avizo software package (Thermo Fisher Scientific). Slice images were converted to 8-bit, and then filters were sequentially applied in order to reduce noise and sharpen boundaries between phases. The filters applied were: non-local means, bilateral, despeckle, and delineate (following the method of Mahmood et al. 2020)^69^. Segmentation was then performed using histogram ranges and min/max thresholding. Identical processing methods were applied to all sample data.

#### DNA extraction

DNA was extracted from 300 µl of homogenised sample using a DNeasy PowerSoil Pro Kit (Qiagen, Manchester, UK), according to the manufacturer’s instructions. Sediment slurries from triplicate samples, taken from citrate-supplied microcosms, were pooled prior to DNA extraction, as individual samples typically yielded insufficient DNA for reliable downstream analysis.

#### 16S rRNA gene sequencing

Sequencing of PCR amplicons of 16S rRNA was conducted with the Illumina MiSeq platform (Illumina, San Diego, CA, USA) targeting the V4 hyper variable region (forward primer, 515 F, 5′-GTGYCAGCMGCCGCGGTAA-3′; reverse primer, 806 R, 5′-GGACTACHVGGGTWTCTAAT-3′) for 2 × 250-bp paired-end sequencing (Illumina^70,71^; Caporaso, 2011; Caporaso et al. 2012). PCR amplification was performed using Roche FastStart High Fidelity PCR System (Roche Diagnostics Ltd, Burgess Hill, UK) in 50 μl reactions under the following conditions: initial denaturation at 95 mMC for 2 min, followed by 36 cycles of 95 °C for 30 s, 55 °C for 30 s, 72 °C for 1 min, and a final extension step of 5 min at 72 °C. The PCR products were purified and normalised to ~20 ng each using the SequalPrep Normalization Kit (Fisher Scientific, Loughborough, UK). The PCR amplicons from all samples were pooled in equimolar ratios. The run was performed using a 4.5 pM sample library spiked with 4.5 pM PhiX to a final concentration of 12% following the method of Schloss and Kozich (2013)^72^.

#### Post-sequencing analysis

A custom bioinformatics pipeline was implemented using a combination of established tools (Cutadapt, SPAdes, PANDAseq, etc.), as previously detailed^73^. Raw sequences were demultiplexed based on barcode identifiers, allowing for up to one mismatch. Quality control and trimming were performed using Cutadapt^74^, FastQC^75^, and Sickle^76^. MiSeq error correction was applied using SPAdes^77^. Forward and reverse reads were merged into full-length sequences using PANDAseq^78^, and chimeric sequences were removed with ChimeraSlayer^79^. Operational taxonomic units (OTUs) were generated using UPARSE^80^, clustered at 97% similarity with USEARCH^81^, and singleton OTUs were excluded. Taxonomic classification was performed using the RDP naïve Bayesian classifier v2.2^82^ in combination with the SILVA SSU 132 database^83^. Rarefaction analysis was conducted in QIIME v1.2 using the original OTU table. Samples with fewer than 5000 sequences were excluded prior to rarefaction. The remaining OTU table was rarefied to the sequencing depth of the sample with the lowest read count. Rarefaction was performed with a step size of 2000 sequences, and 10 iterations were conducted at each step to assess diversity metrics across varying sequencing depths Stacked bar and rarefaction plots were generated in Origin.

## Supplementary information

Supporting Information

## Data Availability

The datasets presented in this study can be found in online repositories. The name of the repository/repositories: NCBI SRA and accession number(s): pending (awaiting release from NCBI).

## References

[1] Iaea. Status A. N. D. Trends In Spent Fuel and radioactive waste management. *IAEA NUCLEAR ENERGY SERIES* No. NP-T-**3**, 1-57 (2018).

[2] Finster, M. & Kamboj, S. International Low Level Waste Disposal Practices and Facilities. Argonne National Laboratory, (2011).

[3] Koťátková, J., Zatloukal, J., Reiterman, P. & Kolář, K. Concrete and cement composites used for radioactive waste deposition. *J. Environ. Radioactivity* **178-179**, 147–155 (2017). 10.1016/j.jenvrad.2017.08.012 28843164

[4] Berner, U. R. Evolution of pore water chemistry during degradation of cement in a radioactive waste repository environment. *Waste Manag.* **12**, 201–219 (1992).

[5] Kuippers, G., Bassil, N. M. & Lloyd, J. R. *Microbial colonization of cementitious geodisposal facilities, and potential “biobarrier” formation*. (Elsevier Inc., 2021).

[6] Small, J. S. & Abrahamsen-Mills, L. *Modeling of microbial processes to support the safety case for nuclear waste disposal*. (Elsevier Inc., 2021).

[7] Butterworth, S. J., Stroes-Gascoyne, S. & Lloyd, J. R. *The microbiology of natural analogue sites*. (Elsevier Inc., 2021).

[8] Rizoulis, A., Milodowski, A. E., Morris, K. & Lloyd, J. R. Bacterial Diversity in the Hyperalkaline Allas Springs (Cyprus), a Natural Analogue for Cementitious Radioactive Waste Repository. *Geomicrobiol. J.* **33**, 73–84 (2016).

[9] Burke, I. T. et al. Biogeochemical Reduction Processes in a Hyper-Alkaline Leachate Affected Soil Profile. *Geomicrobiol. J.* **29**, 769–779 (2012).

[10] Williamson, A. J. et al. Microbial reduction of U(VI) under alkaline conditions: Implications for radioactive waste geodisposal. *Environ. Sci. Technol.* **48**, 13549–13556 (2014). 25231875 10.1021/es5017125

[11] Williamson, A. J. et al. Microbially mediated reduction of Np(V) by a consortium of alkaline tolerant Fe(III)-reducing bacteria. *Mineralogical Mag.* **79**, 1287–1295 (2015).

[12] Bassil, N. M., Bryan, N. & Lloyd, J. R. Microbial degradation of isosaccharinic acid at high pH. *ISME J.* **9**, 310–320 (2015). 25062127 10.1038/ismej.2014.125 PMC4303625

[13] Rout, S. P. et al. Anoxic biodegradation of isosaccharinic acids at alkaline pH by natural microbial communities. *PLoS ONE* **10**, 1–17 (2015). 10.1371/journal.pone.0137682 PMC4569480 26367005

[14] Smith, S. L., Rizoulis, A., West, J. M. & Lloyd, J. R. The Microbial Ecology of a Hyper-Alkaline Spring, and Impacts of an Alkali-Tolerant Community During Sandstone Batch and Column Experiments Representative of a Geological Disposal Facility for Intermediate-Level Radioactive Waste. *Geomicrobiol. J.* **33**, 455–467 (2016).

[15] Byrd, N. et al. Microbial degradation of citric acid in low level radioactive waste disposal. *impact biomineralisation React.* **12**, 1–22 (2021). 10.3389/fmicb.2021.565855 PMC8114274 33995289

[16] Borges, P. H. R. et al. Carbonation durability of blended cement pastes used for waste encapsulation. *Mater. Struct./Materiaux et. Constr.* **45**, 663–678 (2012).

[17] Sun, J. & Simons, S. J. R. Accelerated carbonation study for the prediction of long term performance of the Nirex Reference Vault Backfill in radioactive waste disposal. *AIChE Annual Meeting, Conference Proceedings*, 1-8 (2008).

[18] De Muynck, W., De Belie, N. & Verstraete, W. Microbial carbonate precipitation in construction materials: A review. *Ecol. Eng.* **36**, 118–136 (2010).

[19] Jonkers, H. M. Bacteria-based self-healing concrete. *Heron* **56**, 5–16 (2011).

[20] Vijay, K., Murmu, M. & Deo, S. V. Bacteria based self healing concrete – A review. *Constr. Build. Mater.* **152**, 1008–1014 (2017).

[21] Sellafield Ltd. (Online, 2023).

[22] Glasser, F. P., Marchand, J. & Samson, E. Durability of concrete - Degradation phenomena involving detrimental chemical reactions. *Cem. Concr. Res.* **38**, 226–246 (2008).

[23] Purser, G. et al. Modification to the flow properties of repository cement as a result of carbonation. *Geol. Soc. Spec. Publ.* **415**, 35–46 (2015).

[24] Collier, N. C. et al. Gaseous carbonation of cementitious backfill for geological disposal of radioactive waste: Nirex Reference Vault Backfill. *Appl. Geochem.* **106**, 120–133 (2019).

[25] Reinhardt, H. W. & Jooss, M. Permeability and self-healing of cracked concrete as a function of temperature and crack width. *Cem. Concr. Res.* **33**, 981–985 (2003).

[26] Ranaivomanana, H., Verdier, J., Sellier, A. & Bourbon, X. Sealing process induced by carbonation of localized cracks in cementitious materials. *Cem. Concr. Compos.* **37**, 37–46 (2013).

[27] White-Pettigrew, M. et al. Enhanced Strontium Removal through Microbially Induced Carbonate Precipitation by Indigenous Ureolytic Bacteria. *ACS Earth Space Chem.* **8**, 483–498 (2024). 38533191 10.1021/acsearthspacechem.3c00252 PMC10961847

[28] Rochelle, C. A., Purser, G., Milodowski, A. E. & Wagner, D. Results of laboratory carbonation experiments on Nirex Reference Vault Backfill Cement. (2014).

[29] Rizoulis, A., Steele, H. M., Morris, K. & Lloyd, J. R. The potential impact of anaerobic microbial metabolism during the geological disposal of intermediate-level waste. *Mineralogical Mag.* **76**, 3261–3270 (2012).

[30] Francis, A. J., Dodge, C. J. & Gillow, J. B. Biotransformation of plutonium complexed with citric acid. *Radiochimica Acta* **94**, 731–737 (2006).

[31] Francis, A. J. & Dodge, C. J. Bioreduction of uranium(VI) complexed with citric acid by Clostridia affects its structure and solubility. *Environ. Sci. Technol.* **42**, 8277–8282 (2008). 19068806 10.1021/es801045m

[32] LLW Repository Ltd Waste Acceptance Criteria – Low Level Waste Disposal. *WSC-WAC-LOW* **5**, 1–27 (2016).

[33] L. L. W. Repository Ltd. Inventory. *LLWR/ESC/R(11)10019* (2011).

[34] Rafrafi, Y., Ranaivomanana, H., Bertron, A., Albrecht, A. & Erable, B. Surface and bacterial reduction of nitrate at alkaline pH: Conditions comparable to a nuclear waste repository. *Int. Biodeterior. Biodegrad.* **101**, 12–22 (2015).

[35] Rafrafi, Y. et al. Use of a continuous-flow bioreactor to evaluate nitrate reduction rate of Halomonas desiderata in cementitious environment relevant to nuclear waste deep repository. *Biochemical Eng. J.* **125**, 161–170 (2017).

[36] Durban, N. et al. Nitrate and nitrite reduction at high pH in a cementitious environment by a microbial microcosm. *Int. Biodeterior. Biodegrad.* **134**, 93–102 (2018).

[37] Albina, P. et al. Nitrate and nitrite bacterial reduction at alkaline pH and high nitrate concentrations, comparison of acetate versus dihydrogen as electron donors. *J. Environ. Manag.* **280**, 111859 (2021). 10.1016/j.jenvman.2020.111859 33352382

[38] Durban, N. et al. Nitrate and nitrite reduction activity of activated sludge microcosm in a highly alkaline environment with solid cementitious material. *Int. Biodeterior. Biodegrad.* **151**, 104971 (2020).

[39] Byrd, N. et al. Anaerobic biodegradation of citric acid in the presence of Ni and U at alkaline pH; impact on metal fate and speciation. *Environ. Sci.: Adv.* **2**, 1196–1209 (2023).

[40] Fujita, Y., Taylor, J. L., Wendt, L. M., Reed, D. W. & Smith, R. W. Evaluating the Potential of Native Ureolytic Microbes To Remediate a 90Sr Contaminated Environment. *Environ. Sci. Technol.* **44**, 7652–7658 (2010). 20815389 10.1021/es101752p

[41] Fujita, Y. et al. Strontium incorporation into calcite generated by bacterial ureolysis. *Geochimica et. Cosmochimica Acta* **68**, 3261–3270 (2004).

[42] McCarty, P. L. Thermodynamic Electron Equivalents Model for Bacterial Yield Prediction: Modifications and Comparative Evaluations. *Biotechnol. Bioeng.* **97**, 377–388 (2006). 10.1002/bit.21250 17089390

[43] Rodriguez-Blanco, J. D., Shaw, S., Bots, P., Roncal-Herrero, T. & Benning, L. G. The role of pH and Mg on the stability and crystallization of amorphous calcium carbonate. *J. Alloy. Compd.* **536**, S477–S479 (2012).

[44] Rodriguez-Blanco, J. D., Shaw, S. & Benning, L. G. The kinetics and mechanisms of amorphous calcium carbonate (ACC) crystallization to calcite, via vaterite. *Nanoscale* **3**, 265–271 (2011). 21069231 10.1039/c0nr00589d

[45] Soler, J. M. Thermodynamic Description of the Solubility of C-S-H Gels in Hydrated Portland Cement. 33-33 (2007).

[46] Demir, I., Guzelkucuk, S., Sevim, O., Filazi, A. & Sengul, C. G. Examination of Microstructure of Fly Ash in Cement. 11–13 (2017).

[47] Glasser, F. P. in *(AEA-TECDOC-CD--1701(Companion CD)* Vol. 19 33-38 (International Atomic Energy Agency, 2013).

[48] Saleh, H. M. & Eskander, S. B. *Innovative cement-based materials for environmental protection and restoration*. *INC*, (2020).

[49] Matschei, T., Lothenbach, B. & Glasser, F. P. The role of calcium carbonate in cement hydration. *Cem. Concr. Res.* **37**, 551–558 (2007).

[50] Brettar, I., Christen, R. & Höfle, M. G. Aquiflexum balticum gen. nov., sp. nov., a novel marine bacterium of the Cytophaga-Flavobacterium-Bacteroides group isolated from surface water of the central Baltic Sea. *Int. J. Syst. Evolut. Microbiol.* **54**, 2335–2341 (2004). 10.1099/ijs.0.63255-0 15545480

[51] Chung, B. S. et al. Hydrogenophaga caeni sp. nov., isolated from activated sludge. *Int. J. Syst. Evolut. Microbiol.* **57**, 1126–1130 (2007). 10.1099/ijs.0.64629-0 17473270

[52] Zhang, S. et al. Defluviimonas pyrenivorans sp. nov., a novel bacterium capable of degrading polycyclic aromatic hydrocarbons. *Int. J. Syst. Evolut. Microbiol.* **68**, 957–961 (2018). 10.1099/ijsem.0.002629 29458487

[53] Siddiqi, M. Z. et al. Simplicispira hankyongi sp. nov., a novel denitrifying bacterium isolated from sludge. *Antonie van. Leeuwenhoek, Int. J. Gen. Mol. Microbiol.* **113**, 331–338 (2020). 10.1007/s10482-019-01341-0 31624971

[54] Nixon, S. L., van Dongen, B. E., Boothman, C., Small, J. S. & Lloyd, J. R. Additives in plasticised polyvinyl chloride fuel microbial nitrate reduction at high pH: Implications for nuclear waste disposal. *Front. Environ. Sci.* **6**, 1–10 (2018).

[55] Tang, C. Y. & Yang, Z. *Transmission Electron Microscopy (TEM)*. (Elsevier B.V., 2017).

[56] Willems, A. et al. Hydrogenophaga, a New Genus of Hydrogen-Oxidizing Bacteria That Includes Hydrogenophaga flava comb. nov. (Formerly Pseudomonas flava), Hydrogenophaga palleronii (Formerly Pseudomonas palleronii), Hydrogenophaga pseudoflava (Formerly Pseudomonas pseudoflava and “Pseudomonas carboxy do flava”), and Hydrogenophaga taeniospiralis (Formerly Pseudomonas taeniospiralis). *Int. J. Syst. Evolut. Microbiol.* **39**, 319–333 (1989).

[57] Liu, J. R. et al. Emended description of the genus Trichococcus, description of Trichococcus collinsii sp. nov., and reclassification of Lactosphaera pasteurii as Trichococcus pasteurii comb. nov. and of Ruminococcus palustris as Trichococcus palustris comb. nov. in the lo. *Int. J. Syst. Evolut. Microbiol.* **52**, 1113–1126 (2002). 10.1099/00207713-52-4-1113 12148615

[58] Dai, Y. M. et al. Characterization of trichococcus paludicola sp. Nov. and trichococcus alkaliphilus sp. nov., isolated from a high-elevation wetland, by phenotypic and genomic analyses. *Int. J. Syst. Evolut. Microbiol.* **68**, 99–105 (2018). 10.1099/ijsem.0.002464 29116035

[59] LLW Repository Ltd. (2011). The 2011 Environmental Safety Case: Near Field (LLWR/ESC/R(11)10021).

[60] Madigan, M. T., Martinko, J., Bender, K., Buckley, D. & Stahl, D. *Brock Biology of Microorganisms*. 14 edn, (Pearson, 2015).

[61] Reeder, R. J. et al. Coprecipitation of uranium(VI) with calcite: XAFS, micro-XAS, and luminescence characterization. *Geochimica et. Cosmochimica Acta* **65**, 3491–3503 (2001).

[62] Reeder, R. J. et al. Site-specific incorporation of uranyl carbonate species at the calcite surface. *Geochimica et. Cosmochimica Acta* **68**, 4799–4808 (2004).

[63] Smith, K. F. et al. U(VI) behaviour in hyperalkaline calcite systems. *Geochimica et. Cosmochimica Acta* **148**, 343–359 (2015).

[64] Littlewood, J. L. et al. Mechanism of Enhanced Strontium Uptake into Calcite via an Amorphous Calcium Carbonate Crystallization Pathway. *Cryst. Growth Des.* **17**, 1214–1223 (2017).

[65] Milodowski, A. E., Shaw, R. P. & Stewart, D. I. The Harpur Hill Site: its geology, evolutionary history and a catalogue of materials present. *British Geological Survey Commissioned Report*, 43–43 (2013).

[66] Wilkins, M. J., Livens, F. R., Vaughan, D. J., Beadle, I. & Lloyd, J. R. The influence of microbial redox cycling on radionuclide mobility in the subsurface at a low-level radioactive waste storage site. *Geobiology* **5**, 293–301 (2007).

[67] Parkhurst, D. L. & Appelo, C. A. J. in *U.S. Geological Survey Techniques and Methods* 497-497 (USGS, 2013).

[68] Giffaut, E. et al. Andra thermodynamic database for performance assessment: ThermoChimie. *Appl. Geochem.* **49**, 225–236 (2014).

[69] Mahmood, S. (2020).

[70] Caporaso, J. G. et al. Ultra-high-throughput microbial community analysis on the Illumina HiSeq and MiSeq platforms. *ISME J.* **6**, 1621–1624 (2012). 22402401 10.1038/ismej.2012.8 PMC3400413

[71] Caporaso, J. G. in *Proceedings of the National Academy of Sciences.* 4516–4522.

[72] Kozich, J. J., Westcott, S. L., Baxter, N. T., Highlander, S. K. & Schloss, P. D. Development of a dual-index sequencing strategy and curation pipeline for analyzing amplicon sequence data on the miseq illumina sequencing platform. *Appl. Environ. Microbiol.* **79**, 5112–5120 (2013). 23793624 10.1128/AEM.01043-13 PMC3753973

[73] Foster, L. et al. Identification of algal rich microbial blooms in the Sellafield Pile Fuel Storage Pond and the application of ultrasonic treatment to control the formation of blooms. *Front. Microbiol.* **14**, 2023 (2023). 10.3389/fmicb.2023.1261801 10.3389/fmicb.2023.1261801 PMC10582928 37860139

[74] Martin, M. Cutadapt removes adapter sequences from high-throughput sequencing reads. *2011* **17**, 3 (2011).

[75] Bioinformatics, B. FastQC.

[76] Joshi, N. & Fass, J. *Sickle: A sliding-window, adaptive, quality-based trimming tool for FastQ files (Version 1.33)* 2011).

[77] Nurk, S. et al. 158–170 (Springer Berlin Heidelberg).

[78] Masella, A. P., Bartram, A. K., Truszkowski, J. M., Brown, D. G. & Neufeld, J. D. PANDAseq: paired-end assembler for illumina sequences. *BMC Bioinforma.* **13**, 31 (2012). 10.1186/1471-2105-13-31 PMC3471323 22333067

[79] Haas, B. J. et al. Chimeric 16S rRNA sequence formation and detection in Sanger and 454-pyrosequenced PCR amplicons. *Genome Res* **21**, 494–504 (2011). 21212162 10.1101/gr.112730.110 PMC3044863

[80] Edgar, R. C. Uparse: highly accurate otu sequences from microbial amplicon reads. *Nat. Methods* **10**, 996–998 (2013). 23955772 10.1038/nmeth.2604

[81] Edgar, R. C. Search and clustering orders of magnitude faster than blast. *Bioinformatics* **26**, 2460–2461 (2010). 20709691 10.1093/bioinformatics/btq461

[82] Wang, Q., Garrity, G. M., Tiedje, J. M. & Cole, J. R. Naive Bayesian classifier for rapid assignment of rRNA sequences into the new bacterial taxonomy. *Appl Environ. Microbiol* **73**, 5261–5267 (2007). 17586664 10.1128/AEM.00062-07 PMC1950982

[83] Quast, C. et al. The silva ribosomal rna gene database project: improved data processing and web-based tools. *Nucleic Acids Res* **41**, D590–D596 (2013). 23193283 10.1093/nar/gks1219 PMC3531112

