# Supplementary material for: Microbial degradation of citrate mediates sealing of cement cracks under anaerobic conditions relevant to radioactive waste disposal
Source: Npj Mater Degrad. 2025 Nov 20;9(1):147. doi: 10.1038/s41529-025-00686-4 (PMC12634444; doi:10.1038/s41529-025-00686-4)
Supplement: Supplementary file 1 — Supporting Information [file 41529_2025_686_MOESM1_ESM.docx]

**Supporting Information**

**Microbial degradation of citrate mediates sealing of cement cracks under anaerobic conditions relevant to radioactive waste disposal**

**Natalie Byrd*^1^**, Ananya Singh^1^, Naji M. Bassil^1γ^, , Joe S. Small^1^, Frank Taylor^2^, Christopher Boothman^1^, Dirk L. Engelberg^4^, Sultan Mahmood^4^, Tristan Lowe^5^, Jonathan R. Lloyd^1^, Katherine Morris*^1^

^1^Radioactive waste Disposal and Environmental Remediation National Nuclear User Facility and Williamson Research Facility, Department of Earth and Environmental Sciences, The University of Manchester, Manchester M13 9PL, UK

^2^Nuclear Waste Services, Pelham House, Seascale, Cumbria CA20 1DB, UK

^3^Manchester X-ray Imaging Facility, Photon Science Institute, University of Manchester, Manchester M13 9PL, United Kingdom

^4^Metallurgy & Corrosion, Department of Materials, The University of Manchester, M13 9PL Manchester, UK.

^5^Natural History Museum Abu Dhabi, Jacques Chirac Street, Abu Dhabi, UAE

*^γ^ has moved to Nuclear Waste Services*

*Correspondence: [katherine.morris@manchester.ac.uk](mailto:katherine.morris@manchester.ac.uk) and natalie.byrd@manchester.ac.uk


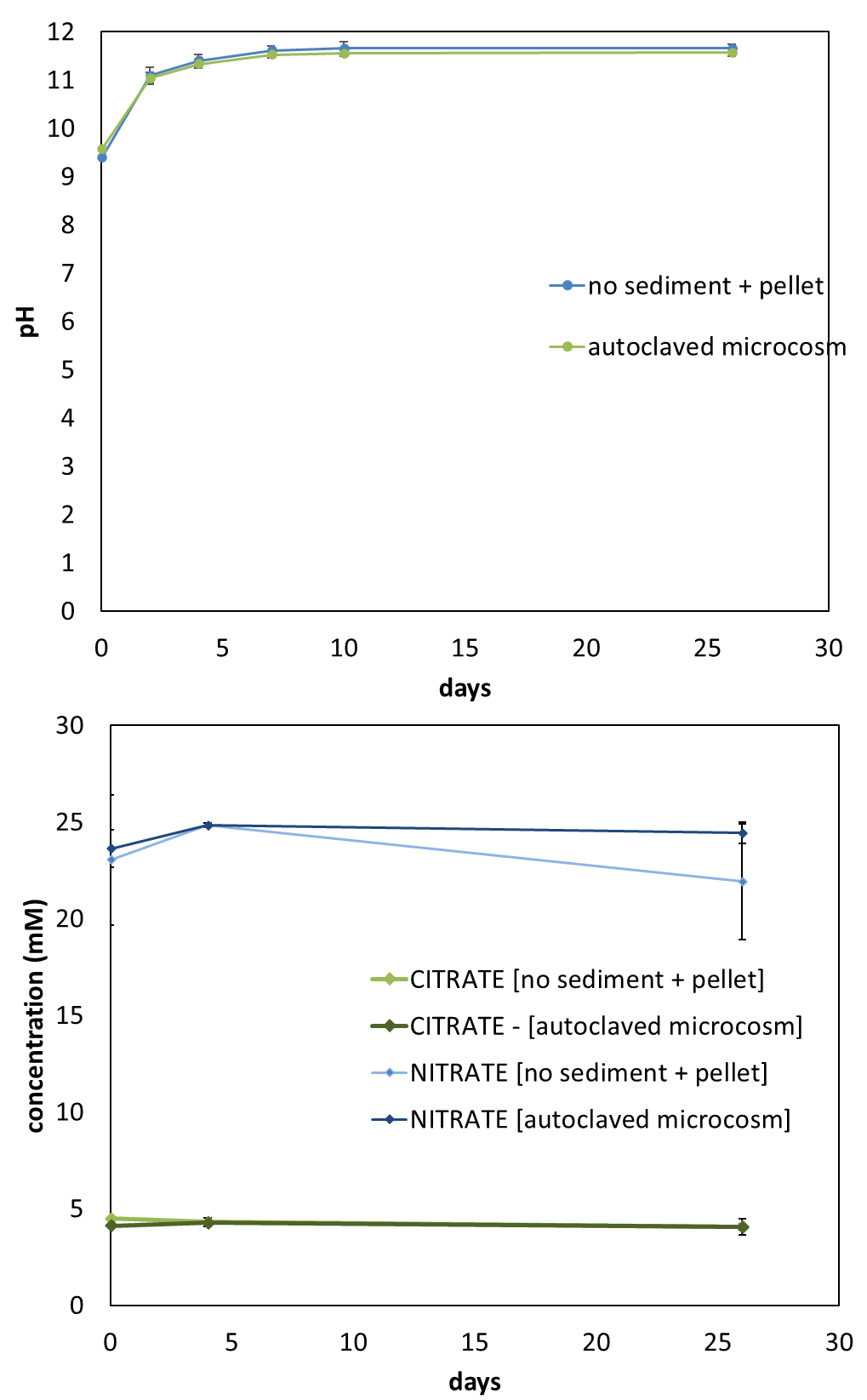


**Figure S1**. Sterile control data: pH (top) and IC (bottom) measurements of citrate and nitrate. Measurements taken from triplicate autoclaved microcosms after 26 days (containing citrate, nitrate, cement pellet, groundwater, and sediment) and triplicate sediment-free control systems (containing citrate, nitrate and cement).

*Table S1. Standard eeq mol-1 values used in electron balance assessment calculations (calculated from standard half equations^1^), plus IC measured ∆(Cit), ∆(NO_3_^-^), and ∆(NO_2_^-^) values and subsequent electron balance assessment. Citrate degradation mM eeq: 70 ± 5.4 (range: 64.6-75.4); NO_3_^-^ + NO_2_^-^ reduction mM eeq: 61 ± 7.4 (range: 53.6-68.4)*

| **Reaction half equation** | **eeq mol^-1^** | | |
| --- | --- | --- | --- |
| Citrate 🡪 CO_2_ | 18 | | |
| NO_3_^-^ 🡪 NO_2_^-^ | 2 | | |
| NO_2_^-^ 🡪 $\frac{1}{2}$N_2_ | 3 | | |
|  |  |  |  |
| *Electron balance assessment* | | |  |
|  | **∆(Cit)** | **∆(NO_3_^-^)** | **∆(NO_2_^-^)** |
| **mM** | 3.9 ± 0.3 | 23 ± 1.9 | 5 ± 2.1 |
|  |  |  |  |
|  | **Cit** | **NO_3_^-^** | **NO_2_^-^** |
| **mM eeq** | 70 ± 5.4 | 46 ± 3.8 | 15 ± 6.3 |

Table S2. Composition of synthetic LLWR groundwater ^2^

| **LLWR synthetic groundwater** | **g L^-1^** |
| --- | --- |
| KCl | 0.006 |
| MgSO_4_.7H_2_O | 0.0976 |
| MgCl_2_.6H_2_O | 0.081 |
| CaCO_3_ | 0.167 |
| NaCl | 0.0094 |
| NaHCO_3_ | 0.212 |
| NaNO_3_ | 0.0275 |

**Figure S2**. PHREEQC input (top) and output (bottom) file, showing expected aqueous speciation of citrate-supplemented microcosm experiments at day 0.


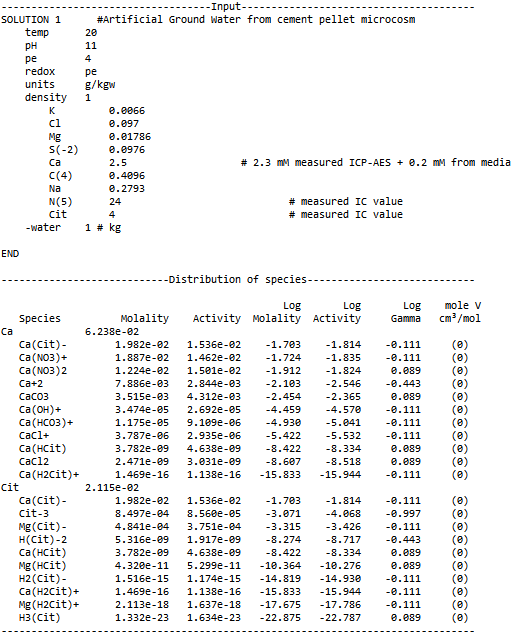

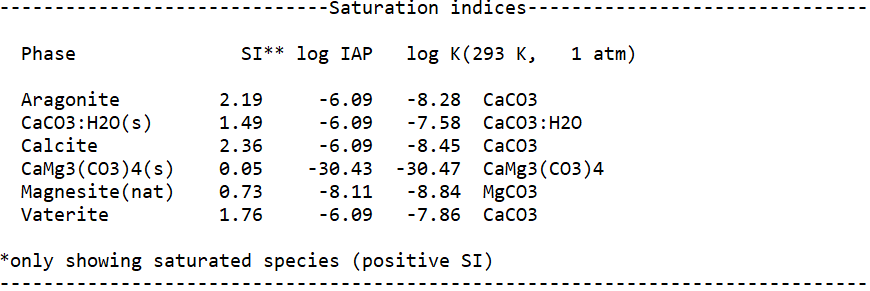


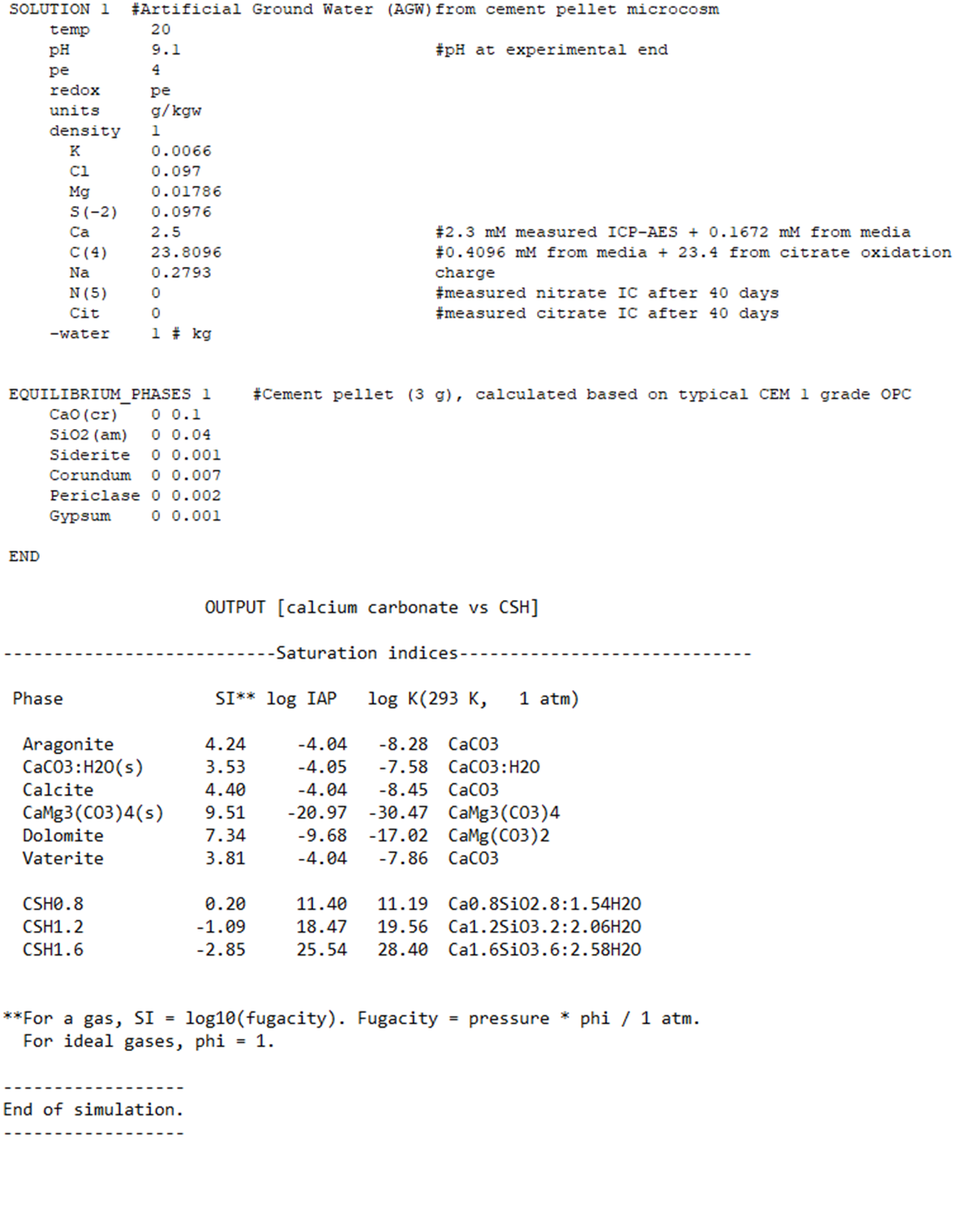


**Figure S3**. PHREEQC input (top) and output (bottom) file, showing expected saturation indices of calcium carbonate phases vs CSH for citrate-supplemented microcosm experiments at day 40.

**Figure S4**. X-ray diffraction spectrum measured from precipitates extracted from the surface of cement pellets (citrate-supplemented microcosm) with the crystal pattern for calcite and quartz superimposed. Crystal pattern matched using Eva v14 against standards from the International Centre for Diffraction Data database.

**Figure S5**. X-ray diffraction spectrum measured from unaltered cement pellets, with various crystal patterns of relevant cement phases superimposed. Crystal pattern matched using Eva v14 against standards from the International Centre for Diffraction Data database*.*


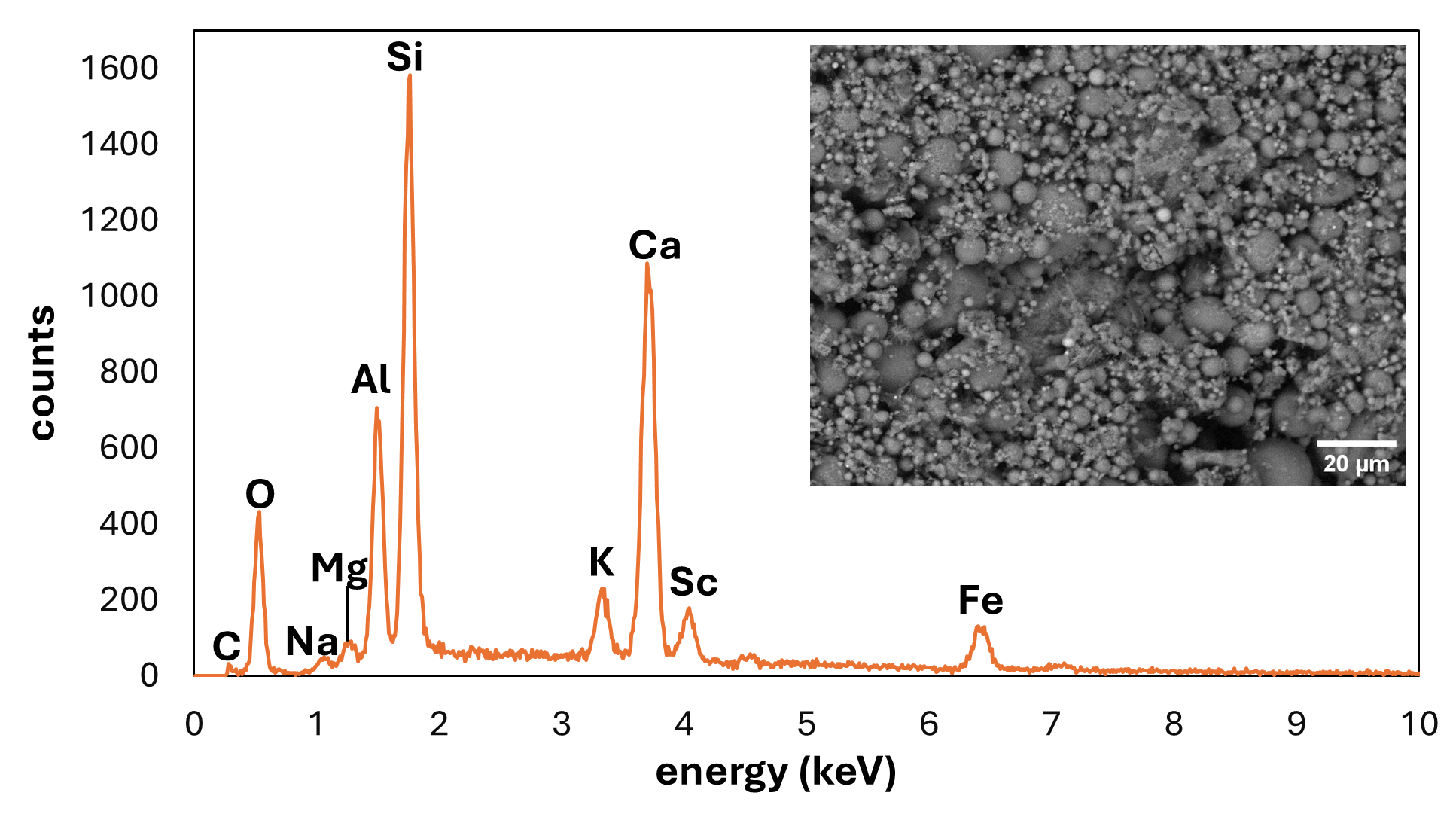


Figure S6. SEM image (inset) and EDX spectra of a washed, soaked and dried cement pellet that has not been incubated in a microcosm.


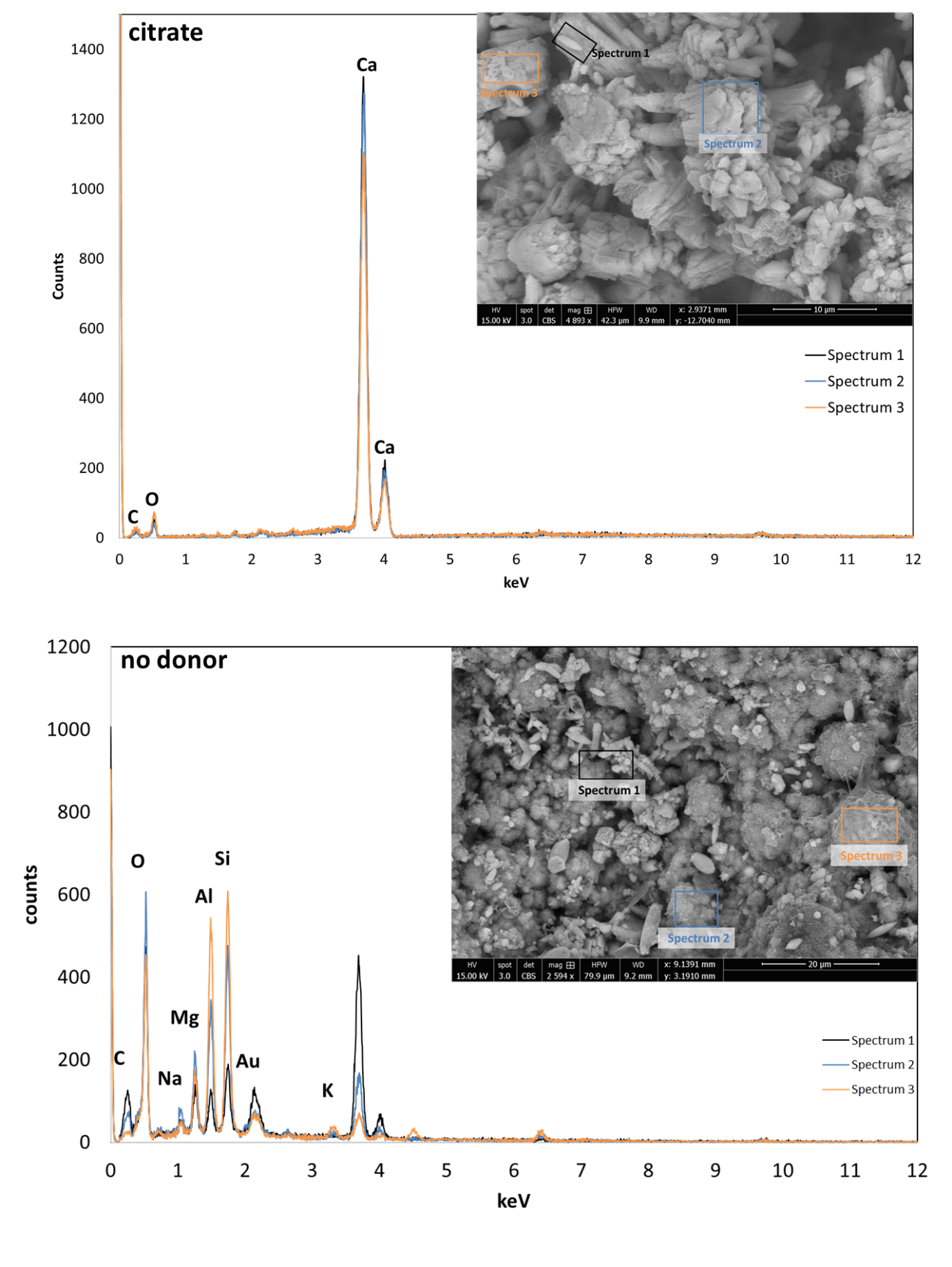


**Figure S7**. SEM images and triplicate EDS scans taken from cement pellets incubated over 40 days in citrate supplemented (top) and no donor (bottom) microcosms.

**Figure S8.** Alpha rarefaction plot showing number or observed species (distinct DNA sequences) in the sediment at day 0 (purple), and after citrate oxidation/nitrate reduction at the experimental endpoint at day 40 (blue)


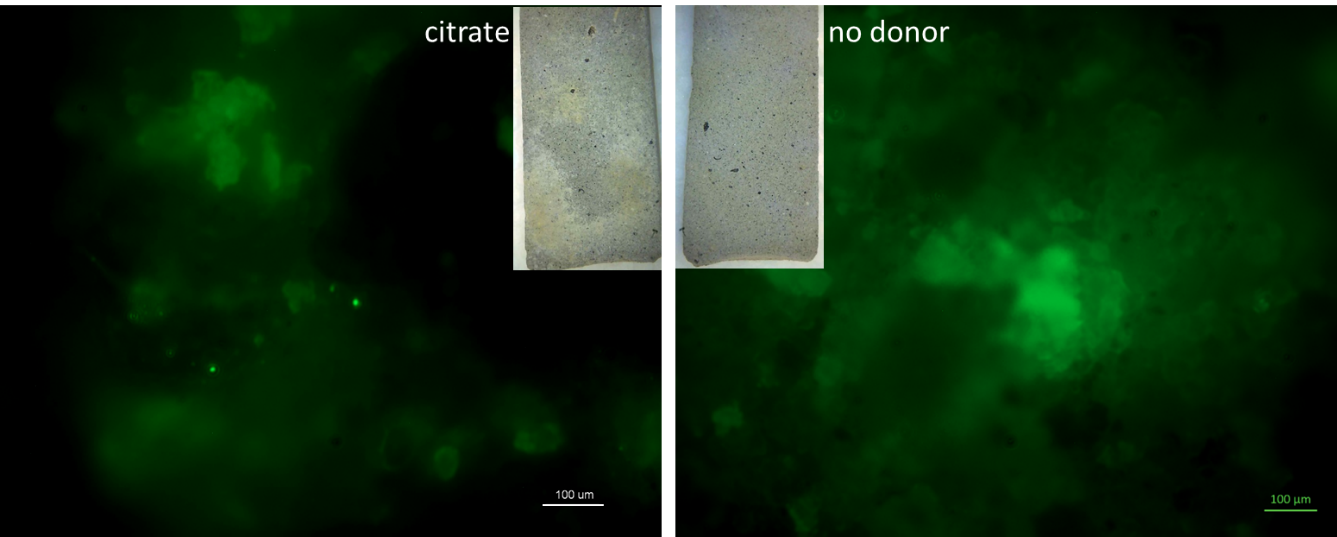


**Figure S9.** Close-up images, and fluorescence microscopy images (after Syto9^TM^ staining) of cement surface from citrate amended microcosm (left) and no-donor control (right). These cement samples were specially prepared by cutting in half to generate flat surfaces to enable fluorescence microscopy imaging, before incubating in microcosms for 40 days (parallel to other microcosm experiments). No microbial colonisation could be observed, presumably fluorescent features visible are auto fluorescent minerals in the cement samples. Scale bars are 100 µM.


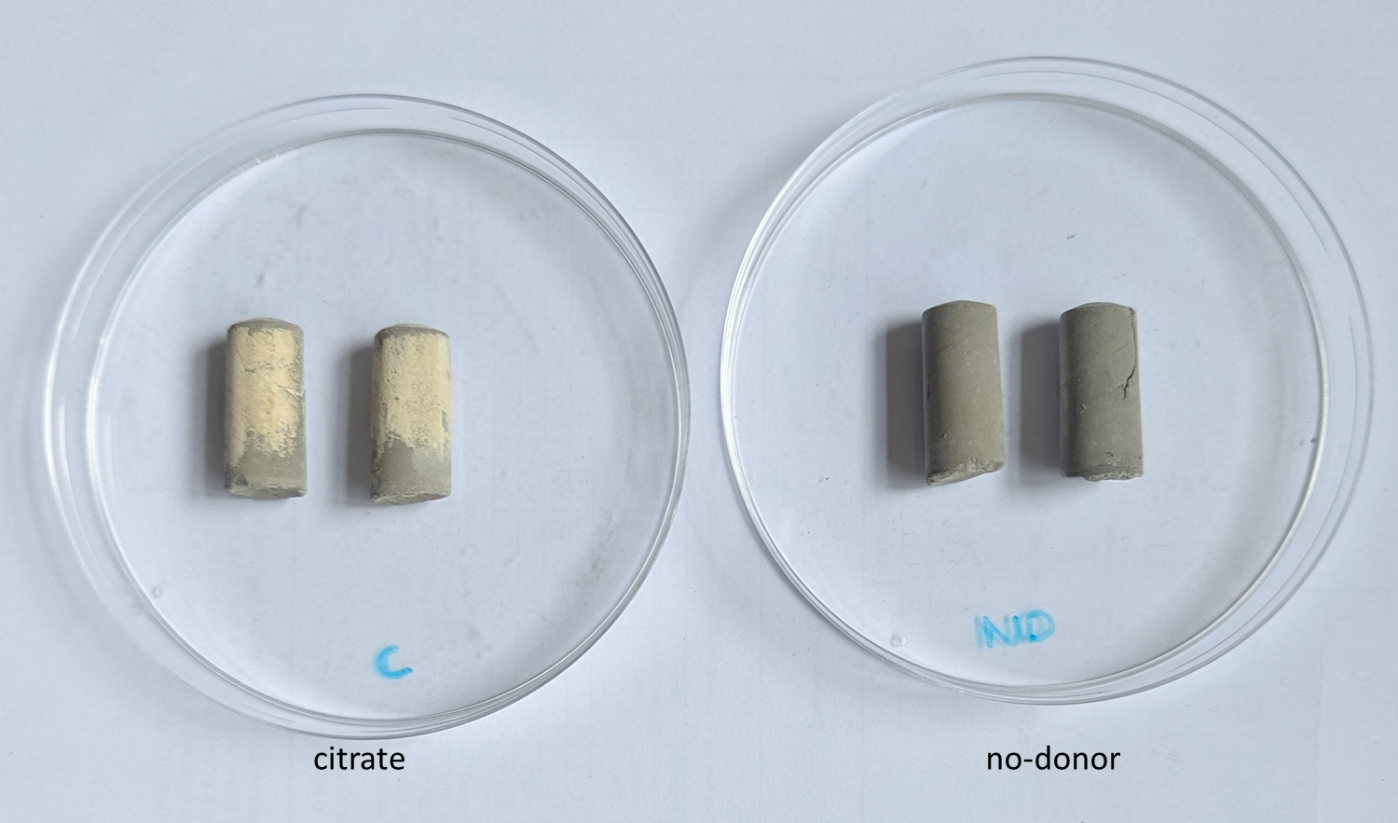


**Figure S10**. Remaining two cement pellets from the citrate-supplemented (left) and no-donor (right) microcosms that were not analysed by XCT. All three pellets in each triplicate microcosm exhibited comparable carbonation behaviour, demonstrating consistency within the experimental sets.


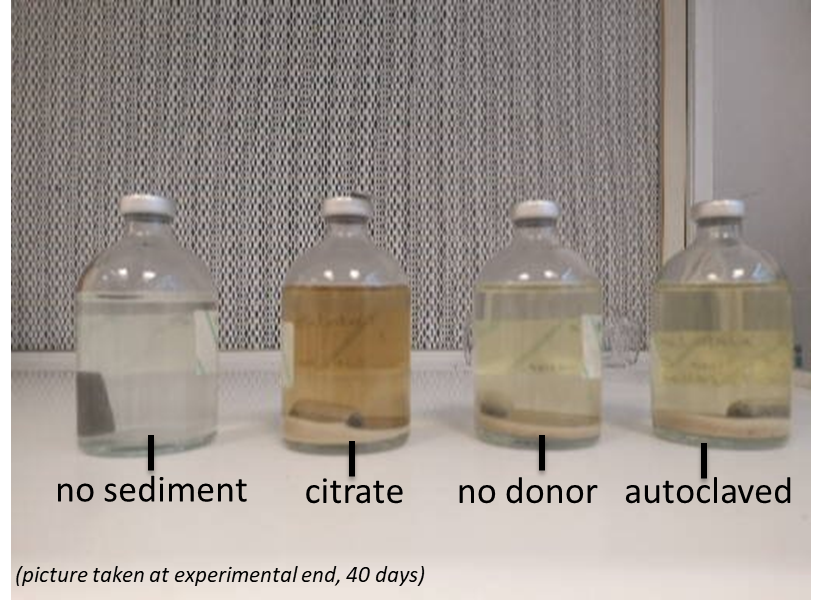


**Figure S11**. Picture showing representative microcosms from each triplicate set, take at day 40 before dismantling.

References

1. McCarty, P.L. Thermodynamic Electron Equivalents Model for Bacterial Yield Prediction: Modifications and Comparative Evaluations. *Biotechnology and Bioengineering* **97**, 377-388 (2006).

2. Wilkins, M.J., Livens, F.R., Vaughan, D.J., Beadle, I. & Lloyd, J.R. The influence of microbial redox cycling on radionuclide mobility in the subsurface at a low-level radioactive waste storage site. *Geobiology* **5**, 293-301 (2007).
